# Multiple Technology Approach Based on Stable Isotope Ratio Analysis, Fourier Transform Infrared Spectrometry and Thermogravimetric Analysis to Ensure the Fungal Origin of the Chitosan

**DOI:** 10.3390/molecules28114324

**Published:** 2023-05-25

**Authors:** Elodie Claverie, Matteo Perini, Rob C. A. Onderwater, Silvia Pianezze, Roberto Larcher, Stéphanie Roosa, Bopha Yada, Ruddy Wattiez

**Affiliations:** 1 MateriaNova ASBL, Avenue Nicolas Copernic 3, 7000 Mons, Belgium; elodie.claverie@materianova.be (E.C.); rob.onderwater@materianova.be (R.C.A.O.); stéphanie.roosa@materianova.be (S.R.); bopha.yada@materianova.be (B.Y.); 2 Fondazione Edmund Mach, Via E. Mach 1, 38098 San Michele all’Adige, Italy; silvia.pianezze@fmach.it (S.P.); roberto.larcher@fmach.it (R.L.); 3 Proteomics and Microbiology Department, University of Mons, Avenue du Champ de Mars 6, 7000 Mons, Belgium; ruddy.wattiez@umons.ac.be

**Keywords:** stable isotope ratio analysis, Fourier transform infrared spectrometry, fungal and crustacean chitosan, origin identification, thermogravimetric analysis

## Abstract

Chitosan is a natural polysaccharide which has been authorized for oenological practices for the treatment of musts and wines. This authorization is limited to chitosan of fungal origin while that of crustacean origin is prohibited. To guarantee its origin, a method based on the measurement of the stable isotope ratios (SIR) of carbon *δ*^13^C, nitrogen *δ*^15^N, oxygen *δ*^18^O and hydrogen *δ*^2^H of chitosan has been recently proposed without indicating the threshold authenticity limits of these parameters which, for the first time, were estimated in this paper. In addition, on part of the samples analysed through SIR, Fourier transform infrared spectrometry (FTIR) and thermogravimetric analysis (TGA) were performed as simple and rapid discrimination methods due to limited technological resources. Samples having *δ*^13^C values above −14.2‰ and below −125.1‰ can be considered as authentic fungal chitosan without needing to analyse other parameters. If the *δ*^13^C value falls between −25.1‰ and −24.9‰, it is necessary to proceed further with the evaluation of the parameter *δ*^15^N, which must be above +2.7‰. Samples having *δ*^18^O values lower than +25.3‰ can be considered as authentic fungal chitosan. The combination of maximum degradation temperatures (obtained using TGA) and peak areas of Amide I and NH_2_/Amide II (obtained using FTIR) also allows the discrimination between the two origins of the polysaccharide. Hierarchical cluster analysis (HCA) and principal component analysis (PCA) based on TGA, FTIR and SIR data successfully distributed the tested samples into informative clusters. Therefore, we present the technologies described as part of a robust analytical strategy for the correct identification of chitosan samples from crustaceans or fungi.

## 1. Introduction

Chitin is a linear polysaccharide polymer composed of D-glucosamine and N-acetyl-D-glucosamine monomers linked by β(1,4) covalent bonds [1]. Chitin is synthesized by a large number of living organisms, such as arthropods, insects, crustaceans, algae, plants and fungi [2]. On industrial scale, the main production comes from crustacean residues (exoskeletons), whereas fungi (cell walls) represent a less used alternative, despite being an abundant chitin source. Chitin is converted into chitosan by deacetylation involving the removal of the acetyl group through alkaline or enzymatic hydrolysis. Both chitin and chitosan are currently intensively used in pharmaceutical, cosmetic, biomedical, biotechnological, agricultural, food, and non-food industries (water treatment, paper and textile industries). These unique polymers are suitable for various applications in many fields due to their excellent biocompatibility, complete biodegradability, low toxicity, non-immunogenic properties and for having a wide range of interesting biological activities, including antimicrobial ones [3,4,5,6,7].

These characteristics make chitosan interesting for winemaking. Wine is a popular beverage consumed worldwide but its quality is influenced by several factors, including the control of *Brettanomyces* yeast population, which is responsible for altering the organoleptic characteristics of the wine. Chitosan has been approved for (1) wine treatment (to reduce heavy metal content, notably iron, lead, cadmium, and copper; to prevent iron haze and copper haze; to reduce possible contaminants, especially ochratoxin A; to reduce undesirable micro-organisms, notably *Brettanomyces* [8]), for (2) wine fining (to reduce turbidity by precipitating particles in suspension and to carry out a treatment to prevent protein haze by the partial precipitation of excess proteinaceous matter [9]) and for (3) must fining (to facilitate settling and clarification and to carry out a treatment to prevent protein haze [10]). However, only fungal chitosan (from *Aspergillus niger* and from *Agaricus bisporus*) is currently authorized for these oenological uses in order to avoid the risks of allergic reaction potentially caused by tropomyosin, the protein contained in shellfish products. In this context, the OIV specified three official methods for the identification of fungal chitosan in the chitosan monograph, i.e., content of residual glucans, viscosity of 1% chitosan solution and settled density [11]. The official methods for chitosan identification have specific limits, since the three mentioned parameters may be falsified to comply with the specifications. 

Chitosan molecular mass distribution, solubility, degree of deacetylation, biological properties and quantity/density of residual saccharides bound to it represent additional techniques tested as tools for the characterisation of this product [12]. However, these procedures, as well as the official ones, are time-consuming and require many different equipment that are not available in all laboratories. For all these reasons, Perini et al. [13] developed a faster and easier method based on the analysis of the stable isotope ratios, expressed in delta, of different elements (^13^C/^12^C, ^15^N/^14^N, ^18^O/^16^O and ^2^H/^1^H). Among these parameters, *δ*^13^C and *δ*^18^O resulted as the most discriminant ones. Although this automated method showed promise as a discriminant tool between fungal and crustacean chitosan, the reduced number of samples analysed and their uncertain origin did not allow Perini et al. [13] to establish a limit value of *δ*^13^C and *δ*^18^O above or below which a sample could be assessed as ‘fungal’ or ‘not fungal’ chitosan.

In this study, for the first time, we analysed chitosan samples of certified origin obtained from crustacean and from *A. niger* and *A. bisporus* strains grown on different substrates with the aim to define threshold limits not only for the isotopic parameters *δ*^13^C and *δ*^18^O but also for *δ*^15^N. We also attempted to develop complementary tools to discriminate between fungal and crustacean chitosan, based on methods widely used in laboratories, namely thermogravimetric analysis (TGA) and Fourier transform infrared (FTIR) spectroscopy. Indeed, FTIR spectroscopy combined with chemometrics has previously been reported as an effective tool in quality control and food origin classification [14,15]. To evaluate the real discriminating ability of the different analytical approaches, we combined them using two different chemometric techniques, principal component analysis (PCA) and hierarchical cluster analysis (HCA).

## 2. Results and Discussion

### 2.1. Characterisation of Fungal and Crustacean Chitosan

#### 2.1.1. Characterisation of Chitosan from Different Origins Using SIR Analysis and Definition of Threshold Limits

Based on the *δ*^13^C and *δ*^15^N found in chitosan samples from crustacean (CC) and from fungal (strain *A. bisporus* and *A. niger* grown on substrate from plants with C3 photosynthetic cycle (FC3) and C4 (FC4)), it was possible to separate not only these two types of products but also to characterise two subclasses (FC3 and FC4) of fungal-derived chitosan. As shown in Table 1, the *δ*^13^C of chitosan from animal exoskeleton (reported as CC-1 to CC-17) ranges from −24.6‰ to −17.2‰ while the *δ*^15^N ranges from −5.7‰ to 0.0‰. With the current dataset, it is not possible to detect statistically significant differences among samples from different exoskeletons (shrimp vs. crab vs. squid) (*p* > 0.05). All the *δ*^13^C and *δ*^15^N found in this study fell within the range of variability reported by Perini et al. for animal chitosan, thus confirming the method validity [13]. 

**Figure 1:**
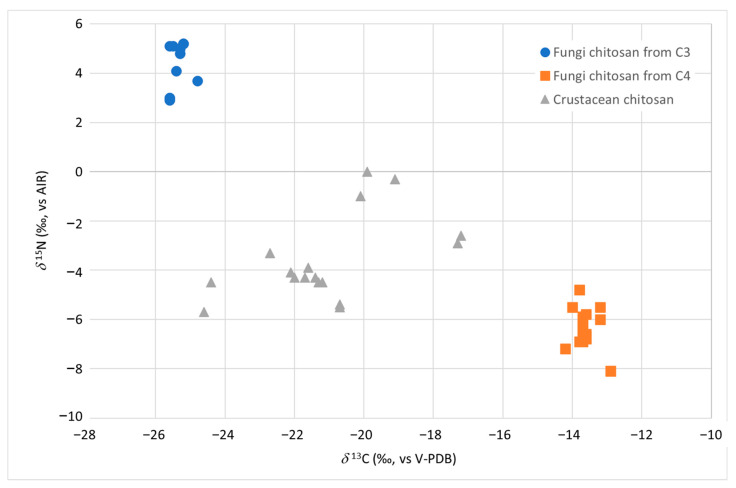
*δ*^13^C vs. *δ*^15^N distribution of chitosan samples from fungi (strain *A. bisporus* and *A. niger* grown on C3 and C4 substrate in blue and orange, respectively) and from crustacean (in grey).

As for fungal chitosan samples (reported as FC3 and FC4 in Table 1), the isotopic values *δ*^13^C and *δ*^15^N allowed to identify not only their fungal origin but also to differentiate them according to the carbon and nitrogen source used by *A. niger* and *A. bisporus* during the growth of their mycelium (Figure 1).

Fungal chitosan could be obtained by *A. niger* mycelium, a by-product of the citric acid synthesis through sugar fermentation carried by *A. niger* strains. Indeed, strains of *A. niger* are used in the production of citric acid since they can use low-cost raw materials (among which the most widespread are beet or cane molasses) and produce higher quantities of citric acid, resulting in a less expensive process [16]. 

This fermentation process involves the use of sugar (such as glucose and sucrose) both in the inoculum of the culture and in the fermentation media supplemented with a limited concentration of phosphate, nitrogen and trace elements. Therefore, the carbon used by the strain during its growth derives almost exclusively from beet or cane sugar and reflects the typical *δ*^13^C of a C3 (from −29 to −25‰) or C4 (between −14 and −12‰) photosynthetic cycle plant [17]. After the separation of the citric acid, the fungal mycelium, figuring as a waste product of citric acid synthesis, can be used as the starting raw material to produce chitin and, therefore, chitosan.

An alternative source used as raw material to produce fungal chitosan is the *A. bisporus* mushroom [18]. It is widely cultivated in Europe and represents about 32% of the world mushroom production [19], but its cultivation gives rise to several by-products, including waste and off-grade mushrooms with no suitable commercial use, whose amount ranges between 5% and 20% of production volume [20]. A strategy to reduce the environmental impact of the agro-food industries is the use of by-products of *A. bisporus* for the production of non-animal chitin and chitosan [21]. Additionally, in this case, the substrates used on an industrial level as a source of carbon for the growth of the fungal mycelium are cane or beer, as already discussed for *A. niger*. 

The ranges of variability of the two isotopic ratios *δ*^13^C and *δ*^15^N were found to be significantly different (*p* < 0.01) between the samples of chitosan from fungi and crustaceans and also between the fungal chitosan from strains grown on substrates of C3 (indicated as FC3) and C4 photosynthetic cycle plants (referred to as FC4) (*p* < 0.01) (Table 1). It was therefore necessary to calculate and report separately in Table 1 the dispersion indexes (e.g., average) for the two types of samples, FC3 and FC4. If the carbon source used by the strain came from C3 sugar (samples FC3), the *δ*^13^C varied from −25.6‰ to −24.8‰, while if it came from C4 sugars (samples FC4), it varied from −14.2‰ to −12.9‰ (Table 1). 

The *δ*^13^C alone resulted to be effective in discriminating between chitosan from crustaceans and from fungi given a C4 source for its growth. On the other hand, a slight overlap between the *δ*^13^C of the chitosan samples from crustacean and those from fungi given a C3 source was observed. In this case, the *δ*^15^N parameter allowed to clearly discriminate (*p* < 0.01) between the two groups, with the product from fungi having (average +4.5‰) higher values than in the crustacean one (average −3.4‰). 

The cultivation of the *A. bisporus* mushroom requires the use of substrates, such as chicken and/or horse manure [22]. Like all animal fertilizers, these products have high *δ*^15^N, related to the trophic effect [23]. The same nitrogen sources with high *δ*^15^N could also be used in *A. niger* strains fermentation. These factors may explain the high *δ*^15^N values found in the FC3 samples. On the other hand, as already observed by Perini et al. [13], the *δ*^15^N alone was unable to discriminate between chitosan from crustaceans and chitosan obtained from fungi using C4 sugars as carbon source. Table 1 shows the threshold limit values (calculated as mean ±2 standard deviation) which can be proposed as authenticity limits. For *δ*^13^C values above −14.2‰ and below −25.1‰, the sample can be considered an authentic fungal chitosan without the need to analyse other parameters. If the value of *δ*^13^C falls between −25.1‰ and −24.9‰, it is necessary to proceed with the evaluation of the parameter *δ*^15^N, which must be above +2.7‰.

The isotopic values *δ*^18^O and *δ*^2^H measured in the samples under study are listed in Table 1. As already reported by Perini et al. [13], the two isotopic parameters are not correlated to each other (R^2^ = 0.3925), as they are differently influenced by the isotopic composition of diet and water. In particular, as reported by Nielson and Bowen, the *δ*^2^H of chitin showed a strong linear correlation with both food and water *δ*^2^H, with approximately 26% of the hydrogen signal reflecting food and approximately 38% reflecting water, while more than 69% of oxygen in chitin exchanged with environmental water and only 10% derived from food [24]. The *δ*^18^O parameter appeared to be the most discriminating one, having the lowest values in chitosan samples from fungi (average +23.5‰) and the highest values in samples from crustaceans (average +28.1‰). The use of carbon sources from C3 or C4 plants cannot be discriminated in the samples of fungal origin based on the *δ*^18^O (*p* > 0.05, Table 1). Therefore, the dispersion parameters (e.g., mean) were calculated considering the whole group of fungal chitosan samples (FC3 + FC4). The *δ*^18^O of chitosan is strictly related to the same value of the water used for the formulation of the fermentative medium. This value is supposed to normally range between −14‰ and −6‰ [25], while it is higher in the ocean water where marine invertebrates live (around 0‰ on average) [26]. This may explain the differences found between the samples of fungal origin (FC3 + FC4 together) and those of crustacean origin (CC). The use of the isotopic parameter *δ*^18^O could be therefore useful to identify the origin of chitosan. Calculating a threshold authenticity limit as mean ±2 standard deviation, a sample from fungi could be considered authentic for values lower than +25.3‰.

#### 2.1.2. Characterisation of Chitosan from Different Origins Using FTIR Analysis

**Figure 2:**
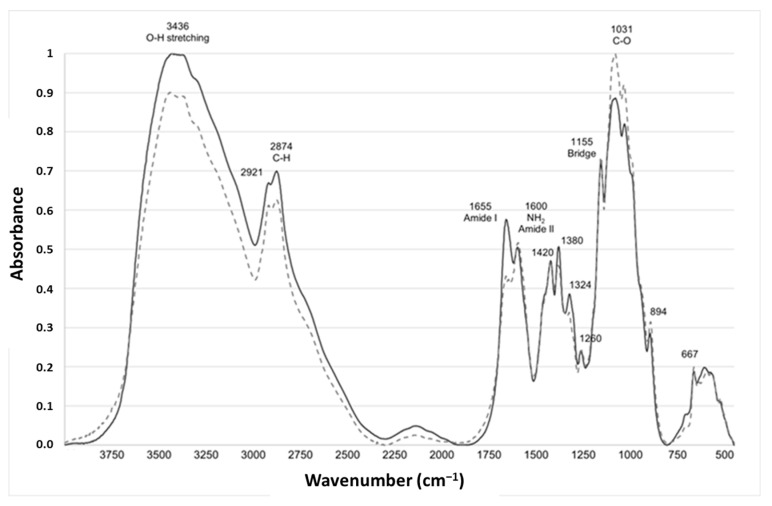
Characteristic infrared absorption spectra of typical fungal (light grey dotted line, FC4-15) and crustacean (solid line dark grey, CC-5) chitosan.

The FTIR is a simple technique performed using equipment that is widely available. FTIR has been used to study the composition and structure of chitin, to distinguish the β-form from the α-one [27,28]. A representative example of spectra from fungal and crustacean chitosan is given in Figure 2. 

Both chitosan spectra showed a series of narrow absorption bands, typical of crystalline polysaccharide samples. The C=O stretching region of the amide moiety of chitosan, evident between 1700 and 1500 cm^−1^, corresponds to the specific signature of α-chitin, the most common form, found in arthropods, including crustaceans, fungi and yeasts, whose amide I band is generally split into two signals at 1660 and 1630 cm^−1^ [29,30].

**Figure 3:**
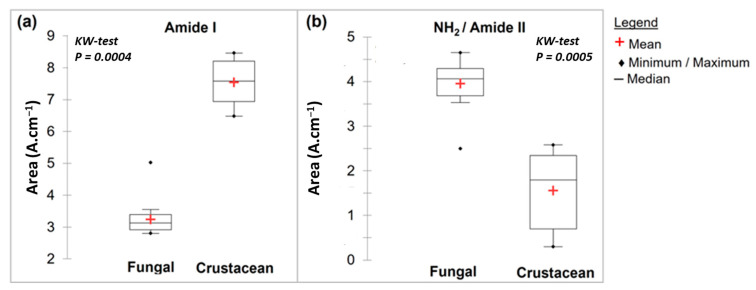
Box plots of FTIR main results with a Kruskal–Wallis evaluation (*p* < 0.05) for fungal and crustacean samples. (**a**,**b**) correspond to Amide I and NH_2_/Amide II peak areas, respectively.

During the N-deacetylation of chitin into chitosan, this amide I band gradually decreases due to the removal of the acetate moiety. In this study, fungal chitosan spectra from *A. niger* are characterized by two typical absorption bands at 1655 cm^−1^ (C=O stretching of amide I band confirming the presence of residual N-acetyl groups) and at 1600 cm^−1^ (N-H bending of the primary amine NH_2_ resulting from the deacetylation of chitin and possible overlap with the N-H bending of the amide II) [31,32]. Hereinafter, these two bands at 1655 and 1600 cm^−1^ will be referred to as Amide I and NH_2_/Amide II, respectively. The IR spectra can be used to distinguish between fungal and crustacean chitosan. The respective areas of these two peaks were therefore calculated to obtain quantitative data to compare the chitosan according to their origin (see Table 1 and Figure 3). It appears that crustacean chitosan presents a larger peak area for amide I than fungal chitosan (on average 7.5 vs. 3.2 A·cm^−1^, respectively—*p* < 0.001) and a smaller peak area for NH_2_/Amide II (on average 1.6 vs. 4.0 A·cm^−1^, respectively—*p* < 0.001).

#### 2.1.3. Characterisation of Chitosan from Different Origins Using TGA Analysis

**Figure 4:**
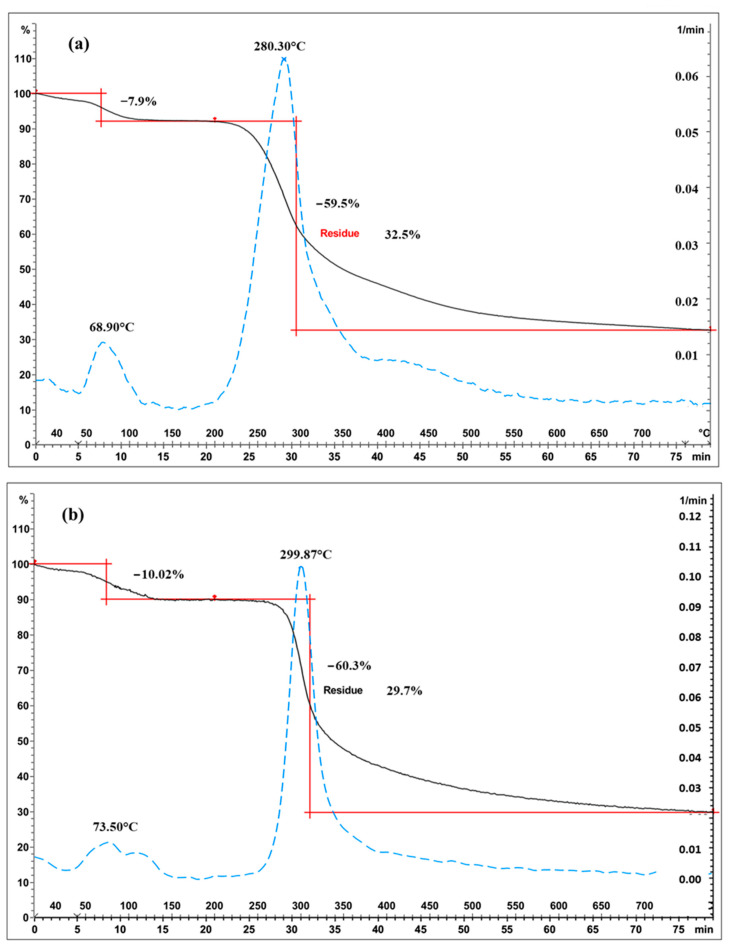
Thermograms of chitosan ((**a**) fungal chitosan FC4-15, (**b**) crustacean chitosan CC-5). Black solid curves represent TGA, blue dotted lines represent the derivatives and red represents the weight loss.

**Figure 5:**
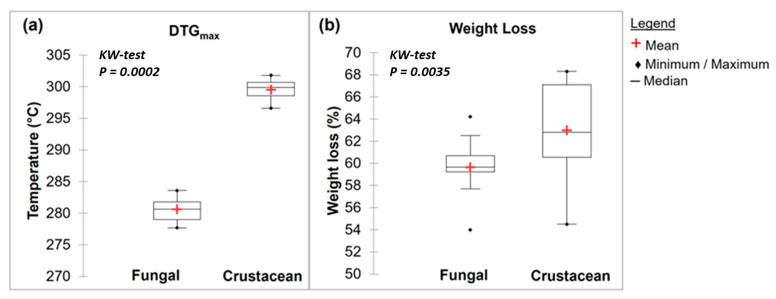
Box plots of TGA main results with a Kruskal–Wallis evaluation (*p* < 0.05) for fungal and crustacean samples (**a**,**b**) correspond to DTGmax and weight loss, respectively.

Representative TGA curves of chitosan are shown in Figure 4a for fungal and Figure 4b for crustacean chitosan. As found in the literature [33,34,35,36], both curves present two degradation stages: the first step occurs around 60 °C (weight loss WL about 5–10%) and is assigned to the evaporation of the residual water because of the strong affinity of polysaccharides for water. Then, the weight of partially deacetylated chitosan remained stable up to 250 °C followed by a rapid substantial loss of weight. The second step occurs around 300 °C (exothermic, WL about 60–70%) and is related to the pyrolytic decomposition of chitosan which is characteristic for the chitosan structure [37,38]. The experimental data obtained from the TGA characterisation of the chitosan samples are summarized in Table 1 and represented in Figure 5. The data show that crustacean chitosan exhibited higher DTGmax than fungal chitosan (average DTGmax value of 299.5 vs. 280.6 °C, respectively—*p* < 0.001). The differences in weight loss are less discriminatory than the maximum degradation temperatures (DTGmax). For this reason, only DTGmax values will be used for the following statistical analysis. It should be noted that few studies focus on the TGA analysis of fungal chitosan, but our results correspond to the DTGmax values reported by Kaya et al., asserting that the low DTGmax value of fungal chitin and chitosan stems from the glucan residues that could not be removed from the chitin structure [29].

### 2.2. Discrimination and Clustering Based on the Origin of Chitosan

Analysis of FTIR and TGA data (Table 1) shows that the origin of the chitosan impacts the intrinsic characteristics of the biopolymer, with significant differences for each parameter studied (KW-test < 0.05). However, the maximum degradation temperature measured using TGA, as well as amide I and NH_2_/amide II peak areas using FTIR seem to be the most promising parameters for determining the origin of chitosan (*p* < 0.001).

**Figure 6:**
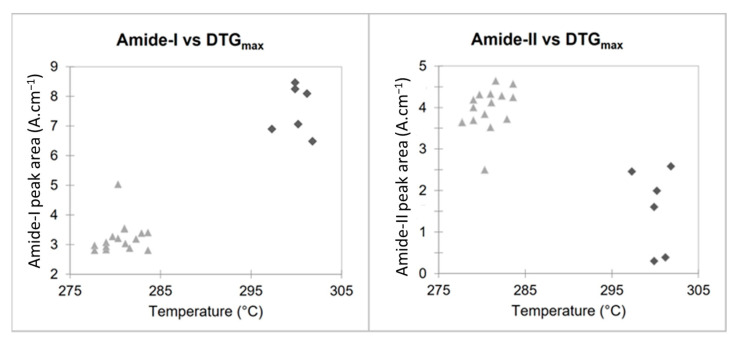
Scatter plots of FTIR and TGA results for fungal (light grey triangle) and crustacean (dark grey losange).

These two parameters, plotted in the scatter plots in Figure 6, show how it is possible to classify the two types of chitosan in a simple and fast way and without the need for specific software. Figure 6 highlights the relationship between the maximum degradation temperatures (obtained using TGA) and areas of the amide I and NH_2_/Amide II peaks (obtained using FTIR). Fungal and crustacean chitosan samples are well clustered into two separate groups. 

**Figure 7:**
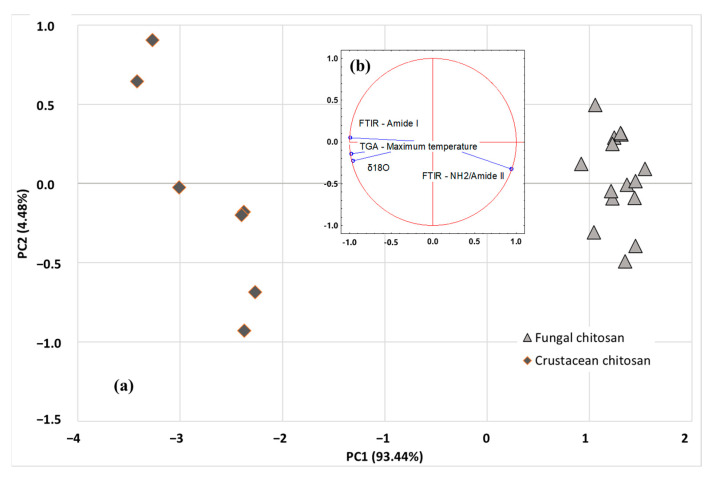
PCA scatter plot (**a**) and related biplots (**b**) of SIR (*δ*^18^O), TGA (DTGmax) and FTIR (Amide I, NH_2_/Amide II) data. Actives observations are represented by light grey triangles (fungal chitosan) or dark grey losange (crustacean chitosan).

More advanced multivariate statistical analysis of the different investigated parameters (SIR, TGA, and FTIR) were obtained using PCA or HCA exploratory methods. Among the isotopic ratios, *δ*^18^O was selected as SIR parameter as it is independent of the carbon source used by the fungi strain, while maximum degradation temperature and ratio of amide I and NH_2_/amide II peak areas were selected as TGA and FTIR parameters, respectively. Unsupervised methods, also named clustering or displays methods, are used to study the data structure and to evaluate whether clustering exists in a dataset. The PCA scatter plot involving the first 2 PCs (i.e., PC1 and PC2) is shown in Figure 7a. The analysis discriminated the origin of chitosan using the first two components: PC1 (93.44%) and PC2 (4.48%), explaining 97.92% of total variance. PCA results revealed that the acquired data points were clearly grouped into two classes (fungal chitosan, represented by light grey triangles or crustacean chitosan, represented by dark grey losange), based on the SIR, TGA and FTIR parameters of the samples. As indicated in Figure 7b, PC1 axis is linked to FTIR parameter amide I, TGA parameter (DTGmax) and *δ*^18^O, whereas PC2 axis is more linked to FTIR parameter NH_2_/Amide II peaks. Once the representative PCs were found, based on sample differentiation/grouping and variance explained, loading analysis is started to find the underlying relationships in the original data structure. The positive factor loadings indicate that the factor will be higher in the positive axis of that PC. For example, for NH_2_/Amide II, a factor loading of 0.942 was obtained with PC1 and, for Amide I, a factor loading of 0.050 was obtained with PC2. It means that the samples located in the right-hand side (i.e., fungi origin) of the graph have higher mean NH_2_/Amide II and Amide I area values than the samples located in the left-hand side (i.e., crustacean origin). Similarly, the negative factor loadings indicate that the factor will be higher in the positive axis of that PC. For example, for DTGmax, a factor loading of −0.976 was obtained with PC1, meaning that the samples located in the right-hand side (i.e., crustacean origin) of the graph have lower mean DTGmax values than the samples located in the left-hand side (i.e., crustacean origin). The study of the regression vectors (see Figure 7b) shows vectors of correlation coefficients between the original variables with each PC-score. 

**Figure 8:**
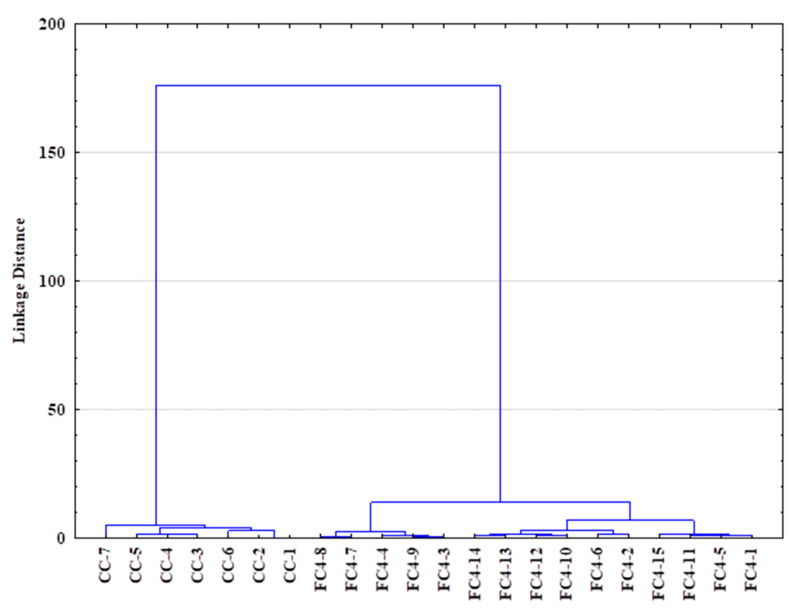
Dendrogram obtained via hierarchical clustering analysis (HCA—Euclidean distances) using SIR, TGA and FTIR data of crustacean (CC) and fungal chitosan sample (FC).

In addition, a hierarchical clustering analysis (HCA) was performed to explore the organization of samples in groups and among groups depicting relationships in tree form (Figure 8). The agglomerative approach was used on the SIR, TGA and FTIR data: the complete linkage method was used for cluster building, and the distance between clusters was computed using the Euclidean method (Ward method, Euclidean distances). Figure 8 shows the similarity dendrogram obtained, highlighting two main clusters: (I) crustacean chitosan and (II) fungal chitosan, thus confirming the PCA results while providing more investigatory outcomes. The obtained HCA dendrogram reveals that fungal samples could be readily distinguished from the crustacean samples. 

## 3. Materials and Methods

### 3.1. Sampling

To build the isotopic database of chitosan, 50 samples of chitosan from different fungal sources and crustacean exoskeleton were obtained from different producers (Table 1). The samples provided by the producers were accompanied by a certificate of analysis highlighting the origin of chitosan (from fungi or crustaceans) and their main characteristics, as well as a description of the production methods, including information about the strain used to produce the fungi (*A. bisporus* and *A. niger*) and the type of substrate used for the growth of the fungal strain.

Since the detailed industrial process is confidential, the exact recipes and the quantities of the individual ingredients have not been provided.

For TGA and FTIR analysis, 15 samples of fungal origin and 7 of animal origin were selected (Table 1). 

In this study, chitosan obtained from fungus is identified as “FC” and more specifically as “FC3” if the substrate used for the growth of the fungi strain was from C3 photosynthetic cycle plants or “FC4” if it was from C4 photosynthetic cycle plants. 

As per the certificate of analysis provided by the suppliers, FC3-1 to FC3-9 are fungal chitosan samples extracted from *A. bisporus.* FC3-10 and FC3-11 and FC4-1 to FC4-22 are fungal chitosan samples extracted from *A. niger.*

Chitosan derived from crustacean is identified as “CC”. CC-1 to CC-17 are crustacean chitosan samples derived from crab, shrimp or squid. A more detailed description of the chitosan products, as well as values of the main molecular characteristics including viscosity in solution and degree ad deacetylation, are reported in Table 1, based on information available from the suppliers. 

### 3.2. Stable Isotope Analysis

The stable isotope ratios of H, C, N and O were measured in pure (95%) bulk lyophilized and ground chitosan, previously washed with a water/alcohol solution (90:10 *v*/*v*). This approach is considerably fast and automated (<10 min for each analysis).

The ^13^C/^12^C and ^15^N/^14^N ratios were measured in one run (around 0.5 mg) using an isotope ratio mass spectrometer (IRMS) (Isoprime Ltd., Cheadle Hulme, UK) following total combustion in an elemental analyser (VARIO CUBE, Elementar Analysensysteme GmbH, Langenselbold, Germany). The ^2^H/^1^H and ^18^O/^16^O ratios were measured in one go (around 0.5 mg) using an IRMS (Finnigan DELTA XP, Thermo Scientific, Waltham, MA, USA) coupled with a pyrolyser (Finnigan TC/EA, high temperature conversion elemental analyser, Thermo Scientific).

Based on the IUPAC protocol [39], the different stable isotope ratios were expressed in the delta scale (*δ*‰) against the international V-PDB (Vienna PeeDee Belemnite) standard according to Equation (1):

(1) $${\delta_{ref}(}^{i}E{/}^{j}E,sample)=\left[\frac{R{(}^{i}E{/}^{j}E,sample)}{R{(}^{i}E{/}^{j}E,ref)}\right]-1$$

where, *ref* is the international measurement standard, *sample* is the analysed sample, and *^i^E*/*^j^E* is the ratio of heavier to lighter isotopes. The delta values were multiplied by 1000 and expressed in “per mil” (‰) units.

The sample analysis was carried out in duplicate. For *δ*^13^C and *δ*^15^N, the isotopic values were calculated against working in-house standards (protein), which were themselves calibrated against international reference materials: fuel oil NBS-22 (*δ*^13^C = −30.03‰), IAEA-CH-6 (IAEA-International Atomic Energy Agency, Vienna, Austria) (*δ*^13^C = −10.45‰) for 13C/12C, L-glutamic acid USGS 40 (U.S. Geological Survey, Reston, VA, USA) (*δ*^13^C = −26.39‰ and *δ*^15^N = −4.52‰) for ^13^C/^12^C and ^15^N/^14^N and potassium nitrate IAEA-NO3 (*δ*^15^N = +4.70‰) for *δ*^15^N.

Both natural chitin and its deacetylation product chitosan contain some strongly adsorbed water that can isotopically exchange its hydrogen with ambient H_2_O and add isotopic noise to the *δ*^2^H of organic hydrogen during the measure. In this study, an equilibration of exchangeable hydrogen in chitin with the H_2_O of the known *δ*^2^H was carried out [40]. 

The *δ*^2^H and *δ*^18^O values were calculated against CBS (Caribou Hoof Standard *δ*^2^H = −157 ± 2‰ and *δ*^18^O = +3.8 ± 0.1‰) and KHS (Kudu Horn Standard, *δ*^2^H = −35.3 ± 1‰ and *δ*^18^O = +20.3 ± 0.2‰) through the creation of a linear equation and by adopting a comparative equilibration procedure [41]. We used these two keratinous standards because of the absence of any international organic reference material with a similar matrix to our samples (chitosan).

The uncertainty of measurements, calculated as standard reproducibility (that we obtain from the analysis of the same sample over time) multiplied for the coverage factor 2, was <0.3‰ for *δ*^13^C and *δ*^15^N analysis, <1‰ for *δ*^18^O, and <3‰ for *δ*^2^H.

### 3.3. Thermogravimetric Analysis

Thermogravimetric analysis (TGA) was performed using a TGA 2 (Mettler-Toledo Garvens GmbH, Giesen, Germany) coupled with the STARe Excellence V16.10 software. A quantity of 3–5 mg of each sample was placed in an open crucible and the temperature was raised from 40 °C to 600 °C at a heating rate of 10 °C per minute.

### 3.4. Fourier Transform Infrared Analysis

The Fourier Transform Infrared (FTIR) analyses were performed using a Spectrum One spectrophotometer (Perkin Elmer, Waltham, MA, USA) operating in transmission mode. FTIR analysis was carried out using the sample dispersed in KBr (1:20 weight ratio). The wave-number range was 4000–400 cm^−1^ and the resolution were 4 cm^−1^, with 12 scans performed on each sample. The system was coupled with the Spectrum V. 3.02.01 software (Perkin Elmer Inc., Wellesley, MA, USA) for further analysis, such as obtaining the area of the characteristic peaks (i.e., 1655 cm^−1^ and 1600 cm^−1^ corresponding to the Amide I and NH_2_/Amide II bands, respectively).

### 3.5. Statistical Analysis

The isotopic data were evaluated using R software, vers. 4.0.4 (R Foundation for Statistical Computing, Vienna, Austria). The FTIR and TGA data were evaluated statistically using XLSTAT (XLSTAT version 2019.2.2, Addinsoft, Paris, France). Data normality was tested (Shapiro–Wilk, *p* < 0.05) and statistical origin differences were checked using the Kruskal–Wallis test (KW-test, *p* < 0.05). 

Principal component analysis (PCA) of samples was carried out to further investigate the differences between the fungal and animal origin of chitosan based on the SIR, FTIR and TGA parameters. Finally, hierarchical clustering analysis (HCA) was carried out based on the SIR, TGA and FTIR data to reveal relationships between samples from the same origin and thus confirm the chitosan origin using XLSTAT.

## 4. Conclusions

In this study, it was demonstrated that *δ*^13^C and *δ*^15^N of the chitosan from fungi are closely related to the substrate used for the growth of the strains (e.g., *A. niger* and *A. bisporus*). Sugars from C3 photosynthetic cycle plants resulted in isotopic ratios different from those from C4 cycle plant, as well as the use of manure instead of urea as a nitrogen source. In both cases, the chitosan from fungi had isotope ratios different from those derived from crustaceans. For the first time, the threshold limits of the isotopic parameters *δ*^13^C, *δ*^15^N and *δ*^18^O of fungal chitosan samples were here defined. 

The data of maximum degradation temperatures (obtained using TGA) combined with those of the peak areas of amide I and NH_2_/Amide II (obtained using FTIR) seemed to allow the differentiation of the two types of chitosan (fungal vs. animal) into well-defined clusters.

Chemometric analyses (PCA and HCA) based on SIR, TGA and FTIR successfully distributed the tested samples into informative clusters, allowing for the differentiation of the samples according to their origin. The results have highlighted a strong similarity between HCA and PCA findings. Chemometric techniques based on SIR, TGA and FTIR provided an efficient, robust methodology for the assessment of chitosan origin. The set of methods provided a multi- approach strategy that appears to be more reliable than the official methods reported in the current monograph of chitosan to prove its authenticity. We therefore present the described technologies as part of an analytical strategy for the correct identification of chitosan samples sourced from crustaceans or fungi. The proposed order of analysis would flow from FTIR analysis, using equipment which is most widely available in labs and has a high throughput, to TGA, which is less widely available and more time consuming, and to SIR, which requires more technological expertise and specialized equipment. Once a sample can clearly be identified with one or more of the technologies, further resources can be spared. Additionally, in any case, upon completion of the full strategy the identification will be without significant doubt for even the most challenging samples.

## Figures and Tables

**Table 1:** Description of the chitosan samples used for SIR, FTIR and TGA analysis, viscosity (cP, 1% in 1% acetic acid) and degree of deacetylation (%) declared and experimental values (mean, SD, min and max, low and high threshold value 95%) of *δ*^13^C (‰, vs. V-PDB), *δ*^15^N (‰, vs. AIR), *δ*^2^H and *δ*^18^O (‰ vs. V-SMOW) isotopic parameters; of the main TGA results (maximum temperature of degradation and weight loss) and of the main FTIR results (amide I and NH_2_/amide II peak areas) obtained from chitosan samples from crustacean (CC) and from fungal (strain *A. bisporus* and *A. niger* grown on substrate C3 (FC3) and C4 (FC4)).

Origin	Fungal Strain/ Crustacean Species	Sample	Viscosity (cP) (1% in 1% Acetic Acid)	Degree of Deacetylation (%)	*δ*^13^C (‰, vs. V-PDB)	*δ*^15^N (‰, vs. V-PDB)	*δ*^18^O (‰, vs. V-SMOW)	*δ*^2^H (‰, vs. V-SMOW)	TGA Parameters		FTIR Parameters	
									Max Temperature of Degradation (DTG_max_ (°C))	Weight Loss (%)	Peak Area (A.cm^−1^)	
											Amide I (1655 cm^−1^)	NH_2_/Amide II (1600 cm^−1^)
*Fungal chitosan*	*A. bisporus*	FC3-1	19.5	84.7	−25.3	5.0	22.0	16.5				
		FC3-2	19.5	84.7	−25.2	5.2	22.4	14.1				
		FC3-3	19.5	84.7	−25.2	5.2	24.3	2.7				
		FC3-4	19.5	84.7	−25.2	5.2	24.3	3.7				
		FC3-5	13.9	84.9	−25.4	4.1	24.1	1.1				
		FC3-6	17.1	84.2	−25.3	4.8	24.2	2.8				
		FC3-7	21.4	83.2	−25.5	5.1	24.3	2.6				
		FC3-8	21.4	83.2	−25.6	5.1	24.4	2.6				
		FC3-9	20.8	84.2	−24.8	3.7	24.8	2.2				
	*A. niger*	FC3-10	nd	nd	−25.6	3.0	24.3	−31.0				
	*A. niger*	FC3-11	nd	nd	−25.6	2.9	24.7	−25.9				
	Mean				−25.3	4.5						
	SD				0.2	0.9						
	Min				−25.6	2.9						
	Max				−24.8	5.2						
	Low Limit 95%				−25.8	2.7						
	High Limit 95%				−24.9	6.3						
	*A. niger*	FC4-1	<15	>70	−13.7	−6.0	23.7	−33.3	282.9	57.7	3.4	3.7
		FC4-2	<15	>70	−13.8	−6.9	24.8	−34.0	281.1	62.5	3.0	4.1
		FC4-3	<15	>70	−13.7	−6.3	23.8	−34.0	279.0	60.7	2.8	4.2
		FC4-4	<15	>70	−13.7	−6.7	23.5	−34.1	279.0	60.2	3.1	3.7
		FC4-5	<15	>70	−13.7	−5.9	23.8	−32.3	283.6	60.7	2.8	4.6
		FC4-6	<15	>70	−13.7	−6.9	23.7	−34.2	281.6	59.5	2.9	4.6
		FC4-7	<15	>70	−13.6	−5.8	23.6	−31.9	277.7	60.6	3.0	3.7
		FC4-8	<15	>70	−13.7	−6.7	24.1	−34.4	277.7	64.2	2.8	3.6
		FC4-9	<15	>70	−13.6	−6.7	24.4	−32.6	279.0	59.8	2.9	4.0
		FC4-10	<15	>70	−13.6	−6.8	22.9	−27.2	280.3	59.5	3.2	3.8
		FC4-11	2.1	80.0	−13.6	−6.7	23.0	−26.1	283.6	54	3.4	4.2
		FC4-12	3.0	82.0	−13.7	−6.3	22.7	−27.5	279.7	59.4	3.3	4.3
		FC4-13	3.1	81.0	−13.7	−6.8	22.9	−28.4	281.0	60.8	3.6	3.5
		FC4-14	2.5	81.0	−13.6	−6.6	23.0	−26.2	281.0	58.7	3.5	4.3
		FC4-15	2.5	85.0	−13.6	−6.8	22.6	−29.9	282.3	59.4	3.2	4.3
		FC4-16	<15	>70	−13.8	−4.8	22.8	7.6				
		FC4-17	<15	>70	−12.9	−8.1	25.1	4.0				
		FC4-18	<15	>70	−14.2	−7.2	22.7	−16.9				
		FC4-19	<15	>70	−14.0	−5.5	22.1	−17.1				
		FC4-20	<15	>70	−13.2	−5.5	23.6	26.6				
		FC4-21	<15	>70	−13.2	−6.0	22.9	18.2				
		FC4-22	<15	>70	−13.7	−6.4	22.7	−28.7				
	Mean				−13.6	−6.4	23.6	−14.6	280.6	59.7	3.2	4.0
	SD				0.3	0.7	0.8	19.6	1.9	2.3	0.5	0.5
	Min				−14.2	−8.1	22.0	−34.4	277.7	54	2.8	2.5
	Max				−12.9	−4.8	25.1	26.6	283.6	64.2	5.0	4.6
	Low Limit 95%				−14.2	−7.8	21.9	−53.8	276.8	55.1	2.2	3
	High Limit 95%				−13.1	−5.0	25.3	24.6	284.4	64.3	4.2	5
*Crustacean chitosan*	Shrimp	CC-1	55.0	83.0	−20.7	−5.4	27.9	20.3	300.2	68.3	7.1	2.0
	Shrimp	CC-2	55.0	83.0	−20.7	−5.5	28.0	22.4	300.2	68.3	7.1	2.0
	Shrimp	CC-3	NA	≥75	−21.3	−4.5	27.9	27.8	299.9	60.3	8.2	0.3
	Shrimp	CC-4	>200	≥75	−21.2	−4.5	28.6	22.9	301.2	60.8	8.1	0.4
	Shrimp	CC-5	<200	nd	−21.4	−4.3	28.7	25.1	299.9	68.1	8.5	1.6
	Crab	CC-6	148.0	90.0	−20.1	−1.0	29.3	30.1	301.8	62.8	6.5	2.6
	Shrimp	CC-7	78.0	89.0	−24.6	−5.7	29.3	26.1	297.3	66.1	6.9	2.5
	Squid	CC-8	3050.0	95.0	−17.3	−2.9	26.5	24.4				
	Crab	CC-9	55.0	91.5	−19.1	−0.3	27.3	24.2				
	Shrimp	CC-10	140.0	90.1	−24.4	−4.5	26.1	3.7				
	Squid	CC-11	2900.0	95.0	−17.2	−2.6	25.4	23.4				
	Shrimp	CC-12	164.0	88.0	−21.7	−4.3	29.6	29.4				
	Crab	CC-13	>400	nd	−19.9	0.0	29.8	30.9				
	Shrimp	CC-14	<200	nd	−22.1	−4.1	28.8	24.8				
	Shrimp	CC-15	>200	≥75	−21.6	−3.9	28.8	22.1				
	Crab	CC-16	20–300	≥75	−22.0	−4.3	28.9	29.4				
	Squid	CC-17	nd	nd	−22.7	−3.3	26.0	8.2				
	Mean				−21.1	−3.6	28.1	23.2	299.5	63	7.5	1.6
	SD				2.0	1.7	1.4	7.2	1.9	5	2.9	1.1
	Min				−24.6	−5.7	25.4	3.7	296.6	54.5	6.5	0.3
	Max				−17.2	0.0	29.8	30.9	301.8	68.3	8.5	2.6
	Low Limit 95%				−25.1	−7.0	25.4	8.8	295.7	53	1.7	−0.6
	High Limit 95%				−17.0	−0.1	30.8	37.7	303.3	73	13.3	3.8

Abbreviation: SD (standard deviation), min (minimum), max (maximum), V-PDB (Vienna Pee Dee Belemnite), V-SMOW (Vienna Standard Mean Ocean Water), nd (not available).

## Data Availability

The raw data presented in this study are available upon request.

## Sample Availability

Samples of chitosan are not available.
